# Radiation Therapy for Adenoid Cystic Carcinoma of the Head and Neck

**DOI:** 10.3390/cancers13246335

**Published:** 2021-12-17

**Authors:** Carlos A. Rodriguez-Russo, Jacqueline C. Junn, Sue S. Yom, Richard L. Bakst

**Affiliations:** 1 Department of Radiation Oncology, Icahn School of Medicine at Mount Sinai, New York, NY 10029, USA; carlos.rodriguez-russo@mountsinai.org; 2 Department of Radiology, Icahn School of Medicine at Mount Sinai, New York, NY 10029, USA; Jacqueline.Junn@mountsinai.org; 3 Department of Radiation Oncology, University of San Francisco, San Francisco, CA 94143, USA; Sue.Yom@ucsf.edu

**Keywords:** adenoid cystic carcinoma, radiotherapy, perineural invasion

## Abstract

**Simple Summary:**

Adenoid cystic carcinoma is a rare histology arising in the head and neck region that has a high propensity for perineural invasion. While surgical resection is the preferred primary treatment modality, adjuvant radiotherapy is often indicated to improve local control. For unresectable disease, definitive radiotherapy can be utilized. Given the predilection for perineural tumor spread, target volumes often must encompass relevant nerve pathways back to the base of skull. Treatment strategies for ACC must therefore balance the disease burden and risk of failure against the morbidity of treatment.

**Abstract:**

Adenoid cystic carcinoma of the head and neck is an uncommon malignancy that can arise in the major or minor salivary glands. Perineural invasion (PNI) is an extremely frequent finding in cases of adenoid cystic carcinoma (ACC) that can be associated with significant patient morbidity and poor prognosis. By contrast, ACC rarely demonstrates lymphovascular space invasion thereby making PNI the major avenue for metastasis and a driver of treatment rationale and design. Radiotherapy is often utilized post-operatively to improve locoregional control or as a primary therapy in unresectable disease. Here we aim to review the role of radiotherapy in the management of this malignancy with a focus on target delineation and treatment regimens in the definitive, recurrent, and metastatic settings.

## 1. Introduction

Adenoid cystic carcinoma (ACC) of the head and neck region, which accounts for 1–2% of all head and neck cancers, is a challenging clinical entity to treat due to its unique clinical and pathologic features and the lack of prospective data guiding ideal treatment approach. This disease is often characterized by a deceivingly indolent presentation followed by perineural invasion (PNI), local recurrence, and metastatic spread. In many cases with nerve invasion, tumor spread along nerve branches can lead to failure at the base of skull—a dreaded complication that is difficult to treat in a salvage setting. In light of the slow but aggressive nature of this rare disease, radiotherapy has emerged as a major treatment modality in the adjuvant and definitive setting to help achieve locoregional control and attempt to reduce risk of spread to nerves and high-risk surrounding tissues. This review aims to summarize the current clinical management of adenoid cystic carcinoma of the head and neck as relevant to the radiation oncologist, with a focus on pathologic risk factors, radiation target delineation, relevant anatomy, and imaging techniques.

## 2. Pathologic Considerations

ACC exhibits varying proportions of three distinct growth patterns referred to as cribriform, tubular, and solid, with cribriform being the most common but the solid type indicating a higher risk of metastasis [1,2]. Perineural invasion (PNI) is frequently seen in pathologic analysis of ACC. Specifically, PNI refers to a histopathologic finding of tumor infiltration into or around nerve tissue; it is important to distinguish this from perineural tumor spread (PNTS), which is clinically or imaging-identified macroscopic extension of a tumor along a nerve. Correlation of PNI with specific ACC histologic subtype is an area of active investigation, with some studies suggesting higher rates of PNI in solid and cribriform subtypes and others showing no correlation [3].

## 3. Radiologic Evaluation of PNTS

Preoperative and pretreatment imaging is important to evaluate for PNTS, defined as the macroscopic tumor extension detectable by imaging along the nerve. Identification of PNTS is important for staging and treatment planning as it may affect the radiation field or tumor resection. However, PNTS evaluation can be challenging due to its intricate anatomy, challenging imaging technique for subtle findings, and the interpreting radiologist’s level of suspicion and knowledge. Additionally, up to 40% of patients with PNTS are asymptomatic [4]. Therefore, an understanding of commonly involved nerves, perineural spread pattern, and optimal imaging techniques is crucial for accurate imaging assessment of disease control. 

## 4. Imaging Techniques

Magnetic resonance imaging (MRI) is the modality of choice to evaluate soft tissue and perineural disease. It has high contrast resolution and allows for superior soft tissue evaluation. MRI has up to 95% sensitivity in detecting PNTS [5]. However, it is important to note that computed tomography (CT) can be helpful in assessing the skull base foramina and is complementary to MRI. 

To evaluate PNTS, it is important to have an optimal imaging protocol that includes the entire course of the nerve with an appropriate field-of-view (FOV). Although 1.5 Tesla (T) is sufficient for evaluation of large cranial nerves, 3T is better at assessing for smaller nerve branches around the ear and parotid regions [6,7]. When imaging for PNTS, images should be thin slices, 3 mm or less, with 3-dimensional acquisition. A FOV should be 16–18 cm, but a smaller FOV may be needed to assess for peripheral and smaller branches.

**Figure 1:**
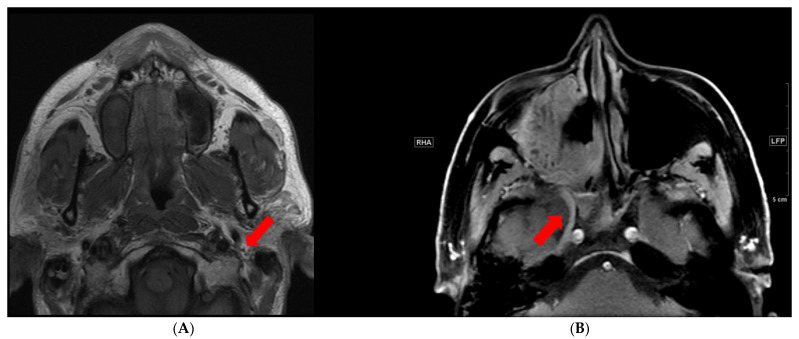
PNTS as seen on MRI. (**A**) Normal T1 hyperintense triangular fat around the facial nerve (arrow). (**B**) Postcontrast fat suppressed T1 image shows abnormally enhancing right V2 (arrow) and the pterygopalatine fossa.

There are certain imaging sequences that are particularly important for PNTS evaluation. T1-weighted pre-contrast images without fat saturation are useful to look for the loss of T1 hyperintense fat that accompanies the T1 hypointense nerves (Figure 1A). This sequence is particularly crucial for evaluation of extracranial cranial nerves [8,9]. Postcontrast T1 should be accompanied by fat suppression. Fat suppression accentuates the abnormal nerve enhancement by eliminating intrinsically bright T1 fat signal that surrounds the nerve (Figure 1B). T2 sequences are important in assessing for edema; this sequence should also have fat suppression to visualize abnormalities. 

More advanced imaging techniques can be utilized to assess nerves, such as MR Neurography. It utilizes special techniques to accentuate the nerves, such as reverse fast imaging with steady state precession, 3D-cranial nerve imaging, high-resolution high-contrast magnetic resonance neurography, and 3D double-echo steady-state with water excitation [10,11]. Specifically, targeted 3T MRI of the nerves has up to 95% sensitivity in detecting PNTS [12]. 

PNTS can be suspected by several imaging features. These include asymmetric enlargement of the nerve, asymmetric enhancement along the nerve, obliteration of the perineural fat planes, and destruction or widening of the neural foramina. Additionally, muscular denervation as a secondary sign can be a clue to suggest a search for PNTS. 

## 5. Relevant Cranial Nerve Anatomy by Primary Site 

ACC frequently occurs in salivary glands throughout the head and neck. It is notorious for centripetal PNTS towards the base of skull. Because of the intricate anatomy of the involved areas, close evaluation of imaging plays an important role during the initial evaluation and in follow-up care; PNTS can be frequently missed by interpreting radiologists [13]. 

### 5.1. Submandibular Gland

When the submandibular gland is involved, the lingual nerve (V3), chorda tympani (VII), and hypoglossal nerve (XII) should be interrogated carefully. Tumor along the lingual nerve can extend cranially through the mandibular nerve via foramen ovale at the skull base. It also receives innervation from the chorda tympani. The lingual nerve and chorda tympani nerve emerge together, which also creates a pathway for tumor to spread along either the trigeminal nerve or facial nerve. Additionally, the hypoglossal nerve is in close proximity to the submandibular gland and is at risk for invasion. 

### 5.2. Parotid Gland

The facial nerve is the most commonly involved nerve for PNTS in the parotid gland. The nerve nuclei are located at the posterior pons. The nerve fibers emerge at the lateral pontomedullary junction and course through the cerebellopontine angle. The facial nerve then enters the temporal bone through the internal auditory canal and continues on as the labyrinthine segment, giving rise to the geniculate ganglion. At the geniculate ganglion, the greater superficial petrosal nerve branches off and travels anteriorly while the remaining facial nerve travels downward at the anterior genu, tympanic segment in the middle ear, posterior genu, and mastoid segment where it gives off the chorda tympani segment. The nerve then exits the temporal bone through the stylomastoid foramen. It is important to scrutinize the stylomastoid foramen for potential PNTS, as the normal fat pad will be lost when there is tumor involvement.

The extra-cranial facial nerve courses through the parotid gland and divides the superficial from the deep lobes. Multiple peripheral branches exist within the parotid gland, which include the posterior auricular, temporal, zygomatic, buccal, marginal mandibular, and cervical nerves. 

The parotid gland also receives parasympathetic fibers from V3 via the auriculotemporal nerve (ATN), which then courses below the foramen spinosum posterior to the mandibular ramus and connects with the VII peripheral branches at the retromandibular region. It is important to note that ATN connects the trigeminal and facial nerves. 

### 5.3. Hard Palate

Hard palate tumors can gain access to V2 via the greater and lesser palatine nerves in the palatine canals that extend superiorly into the pterygopalatine fossa. From here, the tumor can then further extend cranially via the foramen rotundum, cavernous sinus, and Meckel’s cave. When the upper alveolus is involved, the superior alveolar nerve (V2 branch) can also be involved. 

## 6. Nerve Interconnections

It is important to understand common sites where different cranial nerves merge, as these interconnections can serve as a conduit to spread. Tumors can spread between V3 (lingual nerve) and VII (chorda tympani) in the submandibular gland. V2 and VII (vidian nerve) also have connections at the pterygopalatine fossa. V3 (ATN) also serves as a connection to VII, particularly for tumors involving the parotid gland. 

## 7. Imaging Pitfalls

**Figure 2:**
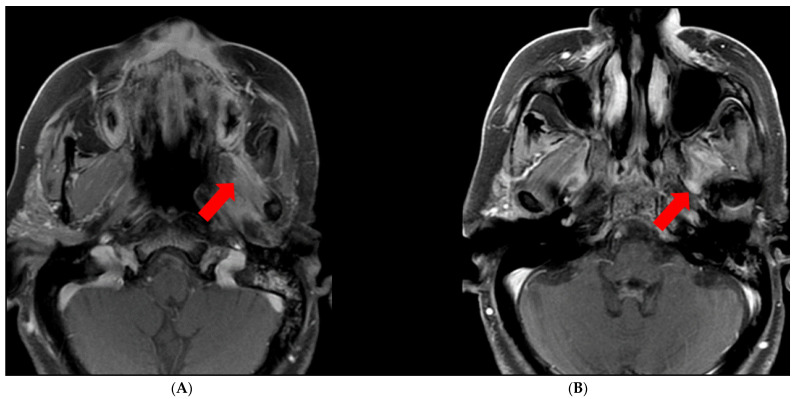
Muscle denervation edema/enhancement. (**A**) Asymmetric enhancement of the left muscles of mastication due to denervation (arrow). (**B**) PNTS along the left V3 (arrow).

There are several technical considerations to keep in mind when assessing for perineural spread. Incomplete fat suppression can occur, especially at the air–bone interface, which can falsely suggest perineural enhancement. It is also important to note that there are parts of the cranial nerves that normally enhance due to perineural venous plexus accompanying the nerves, including the geniculate ganglion, proximal greater superficial petrosal nerve (namely the tympanic and mastoid segments [14]), and the proximal segments of the trigeminal nerves. Denervated muscles demonstrate enhancement and edema, which can have a mass-like appearance and may falsely suggest a mass in that region (Figure 2). Additionally, other entities can mimic PNTS, including infection, inflammation, ischemia, trauma, and demyelinating processes [15]. 

## 8. Rationale for Radiation

Postoperative radiotherapy is nearly always indicated for patients with ACC due to its propensity for local relapse. Traditional indications for postoperative RT include incomplete surgical resection, positive or close margins, and presence of PNI [16,17,18,19,20,21,22,23,24]. In primarily retrospective studies, radiation has demonstrated locoregional control of 36–93% for unresectable or incompletely resected salivary gland tumors, including ACC [25,26,27,28]. Retrospective data on overall survival benefit with postoperative RT is mixed, with one study [29] showing a survival benefit for postoperative RT versus surgery alone (5-year overall survival 82.4% versus 72.5%), while others have shown no survival benefit despite a benefit in locoregional control [25,30,31]. Although radiation is indicated for the vast majority of patients with ACC due to the disease’s propensity for early and late locoregional recurrence and association with perineural invasion, the omission of adjuvant radiotherapy can be considered for highly selected patients with early-stage disease, widely negative surgical margins, and no pathologic evidence of perineural invasion or lymphovascular invasion. Patients electing for observation should be counselled regarding the continued need for careful, long-term clinical follow up to assess for recurrence.

## 9. Radiation Therapy Design

**Figure 3:**
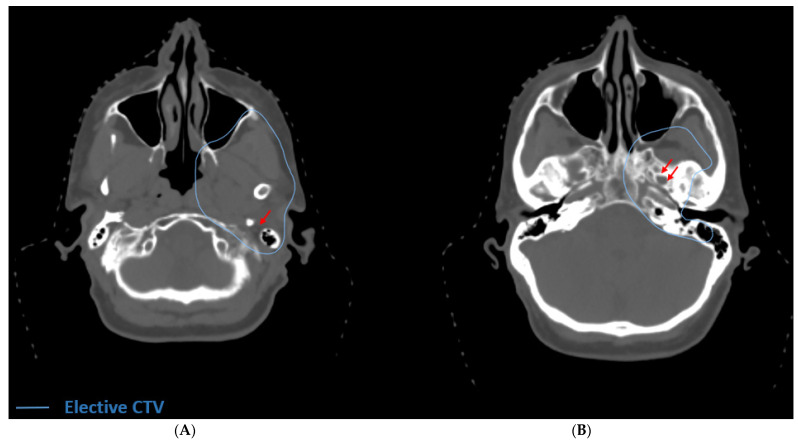
Definitive radiation for unresectable ACC of the deep lobe of the parotid with PNTS. (**A**) The elective volume includes the stylomastoid foramen (red arrow). In this case, there was extension into the parapharyngeal space and infratemporal fossa. (**B**) The elective volume includes the foramen ovale (double red arrows) because of radiographic involvement of V_3_. In this case the elective volume was treated to 56 Gy.

In all surgically resected cases of ACC in which adjuvant radiation therapy is warranted, the primary tumor bed should be covered. The decision of when to electively treat at-risk cranial nerve pathways is more complex. Tracing the CNs back to the base if skull is clinically challenging and can result in increased toxicity; thus it is prudent to consider the balance of potential benefit of elective CN pathway coverage against the toxicity of volume expansion. ACC usually warrants serious consideration of elective coverage of at-risk CN pathways innervating the primary tumor site due to its propensity for PNTS [32,33]. In rare cases of early stage (T1 or T2) ACC of a major salivary gland in which PNI is not observed, treatment of the primary tumor bed alone with margin should be considered. ACC rarely involves the lymphatics [34] and therefore the neck should not routinely be treated unless there is histologically confirmed disease in the neck or a high suspicion based on imaging. Advanced T-stage is associated with an increased risk of nodal involvement, and treatment of the neck can be considered in more advanced cases of this subtype [35]. We have outlined common clinical ACC cases representing a variety of head and neck cases with PNI/PNTS (Table 1). There are previously published contouring guidelines [36,37,38,39,40,41,42] to aid in the target delineation of relevant CN pathways. For ACC arising from the parotid gland with extensive PNI or frank tumor involvement along CN VII, we recommend electively covering the stylomastoid foramen and the proximal course of VII in the temporal bone (Figure 3A) [43,44]. By contrast, in cases of microscopic PNI in early-stage disease of the parotid gland, coverage should only include the stylomastoid foramen and the mastoid segments of VII with the cochlea spared. If there is concern for involvement of the auriculotemporal nerve, it and V_3_ are electively treated up to the foramen ovale (Figure 3B) [45]. 

## 10. Dose Regimen

Just as with volume design, dose selection for ACC must balance treatment toxicity risk against the potential burden of disease and risk of failure at the skull base. Despite the paucity of data guiding dose selection for this disease, dose selection has been previously derived from general principles [36,46]. Postoperatively, a dose of 60 Gy to the primary tumor bed is recommended; this can be escalated to 66 Gy in the setting of close or positive margins [24]. Where elective neural coverage is warranted, a dose of 50–60 Gy is recommended, with dose deescalation to 50 Gy favored for volumes nearest to the base of skull [42,47]. For regions where gross perineural spread is suspected based on operative findings, imaging, or clinical symptoms, dose to involved CN pathways may escalate to 66 Gy. In cases in which there was evidence of lymph node involvement, standard strategies to treat the neck should be applied. In unresectable disease in which radiation therapy is being used definitively, the gross tumor and nerves demonstrating clinical or radiographic involvement receive a dose of 70 Gy, with care taken to deescalate dose beyond involved tissue and nerves. 

Management of the neck in cases which have not been surgically sampled is more challenging. In cases in which the diagnostic imaging suggests involvement of one or both sides of the neck, standard dose and elective volumes should be employed using general principles involved in treating the neck in other head and neck cancers. Fine needle aspirate should be considered before treatment in cases where the imaging is equivocal for nodal involvement to help guide treatment, particularly in the solid subtype. Usually in cases where treatment of the neck is warranted, unilateral neck radiation is sufficient given that the primary tumor is most commonly well lateralized.

## 11. Treatment Related Toxicity

Treatment of the head and neck region with radiation has well documented toxicities [48,49,50]. ACC is unique in that treatment often includes the skull base and CN pathways, which may be fraught with a risk of significant morbidity including permanent blindness, hearing loss, temporal lobe necrosis and cranial neuropathies. Radiation-induced cranial neuropathies can increase over time due to long latency [51,52]; given the long natural history of ACC, this represents a significant concern for patients. Notably, the use of radiosensitizing systemic therapy could enhance radiation toxicity. Depending on the target volumes, patients should be referred pre-treatment to ophthalmology and audiology for baseline visual field and hearing testing, respectively. It is important to recognize that uncontrolled PNTS near the skull base poses a high risk of morbidity from cancer progression [32] and thus high dose radiation is advised despite these risks. An uncontrolled recurrence not only will result in fatality, but also subject the patient to significant end of life morbidity. 

## 12. Particle Therapy

Particle therapies, including proton, neutron, and carbon ion therapies, have been studied within ACC and may allow for further reductions in dose to normal structures compared to conformal photon-based planning. Since treatment of the base of skull is often included in cases of ACC, protons have demonstrated a reduction in the dose to critical structures such as the cochlea, brainstem, and temporal lobe [53]. Protons and other particle therapies should be given extra consideration for unresectable disease where dose escalation is critical [27]. Definitive radiotherapy with particle therapies for unresectable disease has demonstrated a 5-year locoregional control rate of 24–57%, with overall survival of 26.5–87% [54]. The rationale for particle therapy applies even more in the recurrent setting, where it may not be feasible to adequately respect normal tissue constraints using photons. Carbon ion radiotherapy may also hold promise within ACC. Relative to photons, Carbon ion radiotherapy has a relative biologic effectiveness (RBE) of approximately 3, and has demonstrated efficacy and tolerability with a number of centers around the world reporting promising retrospective outcomes in ACC; at least one prospective trial protocol is under recruitment [55,56,57,58]. Neutron therapy has also been utilized within ACC, with promising outcomes in retrospective studies [59,60]. Particle therapy may offer benefits in cases of re-irradiation, discussed below.

## 13. Re-Irradiation

The management of recurrent ACC cancers originating in an irradiated region is complex. Initial surgery of recurrence is ideal and may improve outcomes [61], but unfortunately recurrences related to PNTS are seldom resectable. Re-irradiation may be an option for selected patients, but the therapeutic window of re-irradiation is narrow. Re-irradiation for head and neck cancers in general is far less successful than initial treatment and has a far greater toxicity profile [62]. Given the complexity of the target volumes in upfront treatment, it is not uncommon for such areas of recurrence to have been either omitted or partially treated, allowing for potentially more room for re-irradiation. Obtaining prior radiation records including the target volumes is critical in such cases. Additionally, the patients should receive clinical work up to ensure that there is no distant disease which may change the risk of toxicity the patient is willing to accept. Securing locoregional control with re-irradiation may confer improved long-term quality-of-life compared to uncontrolled locoregional cancer progression, especially when there is PNTS. Irradiation of the involved nerves and elective targeting of adjacent pathways of spread similar to de novo cases is recommended, particularly if these areas were omitted or partially treated in the initial course. Since ACC is often associated with a prolonged natural history, late treatment effects need to be considered and the patient appropriately counseled. Patient selection remains the single most important step in obtaining a beneficial outcome following re-irradiation and often requires a multi-disciplinary team to assist in managing potential toxicities. Time from initial course of radiation to local failure is an important variable to consider when selecting such patients for re-irradiation, with longer intervals being more favorable. When available, particle therapy as discussed above can be considered in these instances due to its physical characteristics, which allow for greater sparing of tissues distal to the radiation target [63]. Re-irradiation with particle therapies has demonstrated a locoregional control of 41–70% with limited follow up in retrospective studies, with overall survival at 2 years of over 60% [64,65,66].

## 14. Concurrent Chemo-Radiotherapy

The role of concurrent chemo-radiation in adenoid cystic carcinoma is the subject of ongoing clinical investigation. For salivary gland malignancies, the clinical trial RTOG 1008 is currently testing whether the addition of cisplatin to standard postoperative radiation for high-risk salivary gland cancers involving the major salivary glands improves survival. High risk factors include pathologic stage T3-4, N1-3 or T1-2, N0 with a close or positive surgical margin. Notably, this randomized phase II/III trial includes high grade ACC (defined as >30% solid component). While we await these results, there are some limited retrospective series that support the use of concurrent chemo-radiotherapy in select patients with adverse pathologic features in salivary gland malignancies [67,68]. In the definitive setting, it is reasonable to extrapolate from other head and neck cancers and consider a concurrent radiosensitizing chemotherapy regimen to maximize tumor control [69]. Multidisciplinary discussion with medical oncology is recommended in such cases.

## 15. Treatment of Metastatic Disease

Adenoid cystic carcinoma has a propensity for early and late spread to the lungs, liver, bones, and brain [70]. Once metastatic, clinical course is highly variable, with some patients experiencing rapid progression. Palliative chemotherapy can be offered for locoregional and distant recurrence in settings where further surgery or radiation would not be possible, but an optimal regimen has not been established [71]. Thus, radiotherapy remains a major modality for palliation and local control in the setting of metastatic disease.

### Management of Oligometastatic Disease

Adenoid cystic carcinoma has a propensity for early but limited distant metastasis, with patients frequently presenting with isolated lung, bone, or liver disease in the absence of other sites of distant spread. The lung is the most common site of distant spread in adenoid cystic carcinoma. For many patients with only one metastatic lung lesion or a limited number of pulmonary metastases within a single lung lobe, metastectomy or lobectomy have been successfully employed for local control [72,73]. For patients who are ineligible for resection or present with multiple pulmonary metastases, however, an effective treatment has yet to be established. Stereotactic Body Radiotherapy (SBRT) has been used for limited lung metastases in ACC, with isolated case reports and at least one retrospective study demonstrating local control of disease with this method [74,75]. Prospective studies investigating the use of SBRT for limited metastasis include the SOLAR trial, a phase II study investigating the use of early SBRT in patients with 1–3 sites of metastatic disease. Radiation in this setting may serve as a promising modality to achieve local control in limited metastatic disease.

## 16. Conclusions

While adenoid cystic carcinoma of the salivary glands is an uncommon malignancy, it may be frequently encountered by radiation oncologists who regularly treat head and neck cancer. Radiotherapy is often utilized post-operatively to improve locoregional control but can serve as a primary therapy in unresectable disease. PNI is a nearly ubiquitous pathologic finding associated with ACC that carries significant clinical implications. Appropriately designed postoperative radiation therapy regimens can improve local control and reduce the risk of hard-to-salvage base of skull failures. For unresectable disease, high radiation doses are required and may risk late onset local toxicities. Treatment regimens for ACC must balance disease burden, the risk of subsequent inoperable disease relapse, and the risk of acute and late toxicity.

## Figures and Tables

**Table 1:** Cranial nerves at risk based on ACC primary site.

Primary ACC Tumor Site	Cranial Nerves at Risk	Origin at Base of Skull	Additional Cranial Nerves at Risk via Inter-Nerve Connections
Submandibular Gland	V_3_	Foramen ovale	VII, via chorda tympani (rarely included in elective volumes as involvement is rare)
	XII (deep lobe involvement)	Hypoglossal canal	
Parotid Gland	VII	Stylomastoid foramen	V_3_, via auriculotemporal nerve
Hard Palate	V_2_	V_2_: foramen rotundum	VII, via greater superficial petrosal nerve and vidian nerve

## Abbreviations

ACC	Adenoid cystic carcinoma
ATN	Auriculotemporal nerve
CN	Cranial nerve
CT	Computed tomography
FOV	Field-of-view
MRI	Magnetic resonance imaging
PNI	Perineural invasion
PNTS	Perineural tumor spread
RT	Radiation therapy
SBRT	Stereotactic body radiotherapy

## References

[1] Azumi N., Battifora H. (1987). The cellular composition of adenoid cystic carcinoma. An immunohistochemical study. Cancer.

[2] Ishida E., Ogawa T., Rokugo M., Ishikawa T., Wakamori S., Ohkoshi A., Usubuchi H., Higashi K., Ishii R., Nakanome A. (2020). Management of adenoid cystic carcinoma of the head and neck: A single-institute study with over 25-year follow-up. Head Face Med..

[3] Barrett A.W., Speight P.M. (2009). Perineural invasion in adenoid cystic carcinoma of the salivary glands: A valid prognostic indicator?. Oral Oncol..

[4] Nemec S.F., Herneth A.M., Czerny C. (2007). Perineural tumor spread in malignant head and neck tumors. Top. Magn. Reson. Imaging.

[5] Caldemeyer K.S., Mathews V.P., Righi P.D., Smith R.R. (1998). Imaging features and clinical significance of perineural spread or extension of head and neck tumors. Radiographics.

[6] Penn R., Abemayor E., Nabili V., Bhuta S., Kirsch C. (2010). Perineural invasion detected by high-field 3.0-T magnetic resonance imaging. Am. J. Otolaryngol..

[7] Gandhi M.R., Panizza B., Kennedy D. (2011). Detecting and defining the anatomic extent of large nerve perineural spread of malignancy: Comparing targeted MRI with the histologic findings following surgery. Head Neck.

[8] Ong C.K., Chong V.F. (2010). Imaging of perineural spread in head and neck tumours. Cancer Imaging.

[9] Curtin H.D. (2004). Detection of perineural spread: Fat suppression versus no fat suppression. Am. J. Neuroradiol..

[10] Wu W., Wu F., Liu D., Zheng C., Kong X., Shu S., Li D., Kong X., Wang L. (2020). Visualization of the morphology and pathology of the peripheral branches of the cranial nerves using three-dimensional high-resolution high-contrast magnetic resonance neurography. Eur. J. Radiol..

[11] Van der Cruyssen F., Croonenborghs T.M., Hermans R., Jacobs R., Casselman J. (2021). 3D Cranial Nerve Imaging, a Novel MR Neurography Technique Using Black-Blood STIR TSE with a Pseudo Steady-State Sweep and Motion-Sensitized Driven Equilibrium Pulse for the Visualization of the Extraforaminal Cranial Nerve Branches. Am. J. Neuroradiol..

[12] Baulch J., Gandhi M., Sommerville J., Panizza B. (2015). 3T MRI evaluation of large nerve perineural spread of head and neck cancers. J. Med. Imaging Radiat. Oncol..

[13] Ginsberg L.E. (2002). Reinterpretation of head and neck scans: Massive can of worms or call to action?. Am. J. Neuroradiol..

[14] Nemzek W.R., Hecht S., Gandour-Edwards R., Donald P., McKennan K. (1998). Perineural spread of head and neck tumors: How accurate is MR imaging?. Am. J. Neuroradiol..

[15] Maroldi R., Farina D., Borghesi A., Marconi A., Gatti E. (2008). Perineural tumor spread. Neuro. Imaging Clin. N. Am..

[16] Bur A.M., Lin A., Weinstein G.S. (2016). Adjuvant radiotherapy for early head and neck squamous cell carcinoma with perineural invasion: A systematic review. Head Neck.

[17] Chatzistefanou I., Lubek J., Markou K., Ord R.A. (2017). The role of perineural invasion in treatment decisions for oral cancer patients: A review of the literature. J. Cranio-Maxillofac. Surg..

[18] Fagan J.J., Collins B., Barnes L., D’Amico F., Myers E.N., Johnson J.T. (1998). Perineural invasion in squamous cell carcinoma of the head and neck. Arch. Otolaryngol. Head Neck Surg..

[19] Tai S.K., Li W.Y., Yang M.H., Chang S.Y., Chu P.Y., Tsai T.L., Wang Y.F., Chang P.M. (2012). Treatment for T1-2 oral squamous cell carcinoma with or without perineural invasion: Neck dissection and postoperative adjuvant therapy. Ann. Surg. Oncol..

[20] Aivazian K., Ebrahimi A., Low T.H., Gao K., Clifford A., Shannon K., Clark J.R., Gupta R. (2015). Perineural invasion in oral squamous cell carcinoma: Quantitative subcategorisation of perineural invasion and prognostication. J. Surg. Oncol..

[21] Lanzer M., Gander T., Kruse A., Luebbers H.T., Reinisch S. (2014). Influence of histopathologic factors on pattern of metastasis in squamous cell carcinoma of the head and neck. Laryngoscope.

[22] Brandwein-Gensler M., Smith R.V., Wang B., Penner C., Theilken A., Broughel D., Schiff B., Owen R.P., Smith J., Sarta C. (2010). Validation of the histologic risk model in a new cohort of patients with head and neck squamous cell carcinoma. Am. J. Surg. Pathol..

[23] Rahima B., Shingaki S., Nagata M., Saito C. (2004). Prognostic significance of perineural invasion in oral and oropharyngeal carcinoma. Oral. Surg. Oral. Med. Oral. Pathol. Oral. Radiol. Endod..

[24] Garden A.S., Weber R.S., Morrison W.H., Ang K.K., Peters L.J. (1995). The influence of positive margins and nerve invasion in adenoid cystic carcinoma of the head and neck treated with surgery and radiation. Int. J. Radiat. Oncol. Biol. Phys..

[25] Balamucki C.J., Amdur R.J., Werning J.W., Vaysberg M., Morris C.G., Kirwan J.M., Mendenhall W.M. (2012). Adenoid cystic carcinoma of the head and neck. Am. J. Otolaryngol..

[26] Douglas J.G., Laramore G.E., Austin-Seymour M., Koh W., Stelzer K., Griffin T.W. (2000). Treatment of locally advanced adenoid cystic carcinoma of the head and neck with neutron radiotherapy. Int. J. Radiat. Oncol. Biol. Phys..

[27] Pommier P., Liebsch N.J., Deschler D.G., Lin D.T., McIntyre J.F., Barker F.G., Adams J.A., Lopes V.V., Varvares M., Loeffler J.S. (2006). Proton beam radiation therapy for skull base adenoid cystic carcinoma. Arch. Otolaryngol. Head Neck Surg..

[28] Gentile M.S., Yip D., Liebsch N.J., Adams J.A., Busse P.M., Chan A.W. (2017). Definitive proton beam therapy for adenoid cystic carcinoma of the nasopharynx involving the base of skull. Oral Oncol..

[29] Lee A., Givi B., Osborn V.W., Schwartz D., Schreiber D. (2017). Patterns of care and survival of adjuvant radiation for major salivary adenoid cystic carcinoma. Laryngoscope.

[30] Choi Y., Kim S.B., Yoon D.H., Kim J.Y., Lee S.W., Cho K.J. (2013). Clinical characteristics and prognostic factors of adenoid cystic carcinoma of the head and neck. Laryngoscope.

[31] Shen C., Xu T., Huang C., Hu C., He S. (2012). Treatment outcomes and prognostic features in adenoid cystic carcinoma originated from the head and neck. Oral Oncol..

[32] Catalano P.J., Sen C., Biller H.F. (1995). Cranial neuropathy secondary to perineural spread of cutaneous malignancies. Am. J. Otol..

[33] Morris J.G., Joffe R. (1983). Perineural spread of cutaneous basal and squamous cell carcinomas. The clinical appearance of spread into the trigeminal and facial nerves. Arch. Neurol..

[34] Min R., Siyi L., Wenjun Y., Ow A., Lizheng W., Minjun D., Chenping Z. (2012). Salivary gland adenoid cystic carcinoma with cervical lymph node metastasis: A preliminary study of 62 cases. Int. J. Oral. Maxillofac. Surg..

[35] Megwalu U.C., Sirjani D. (2017). Risk of Nodal Metastasis in Major Salivary Gland Adenoid Cystic Carcinoma. Otolaryngol. Head Neck Surg..

[36] Ko H.C., Gupta V., Mourad W.F., Hu K.S., Harrison L.B., Som P.M., Bakst R.L. (2014). A contouring guide for head and neck cancers with perineural invasion. Pract. Radiat. Oncol..

[37] Anwar M., Yu Y., Glastonbury C.M., El-Sayed I.H., Yom S.S. (2016). Delineation of radiation therapy target volumes for cutaneous malignancies involving the ophthalmic nerve (cranial nerve V-1) pathway. Pract. Radiat. Oncol..

[38] Mourad W., Hu K.S., Harrison L.B. Cranial Nerves IX-XII Contouring Atlas for Head and Neck Cancer. https://www.rtog.org/LinkClick.aspx?fileticket=B7fuSx-B1GU%3d&tabid=229.

[39] Mourad W.F., Young B.M., Young R., Blakaj D.M., Ohri N., Shourbaji R.A., Manolidis S., Gamez M., Kumar M., Khorsandi A. (2013). Clinical validation and applications for CT-based atlas for contouring the lower cranial nerves for head and neck cancer radiation therapy. Oral Oncol..

[40] Gluck I., Ibrahim M., Popovtzer A., Teknos T.N., Chepeha D.B., Prince M.E., Moyer J.S., Bradford C.R., Eisbruch A. (2009). Skin cancer of the head and neck with perineural invasion: Defining the clinical target volumes based on the pattern of failure. Int. J. Radiat. Oncol. Biol. Phys..

[41] Biau J., Dunet V., Lapeyre M., Simon C., Ozsahin M., Gregoire V., Bourhis J. (2018). Practical clinical guidelines for contouring the trigeminal nerve (V) and its branches in head and neck cancers. Radiother. Oncol..

[42] Bakst R.L., Glastonbury C.M., Parvathaneni U., Katabi N., Hu K.S., Yom S.S. (2019). Perineural Invasion and Perineural Tumor Spread in Head and Neck Cancer. Int. J. Radiat. Oncol. Biol. Phys..

[43] Leonetti J.P., Marzo S.J., Agarwal N. (2008). Adenoid cystic carcinoma of the parotid gland with temporal bone invasion. Otol. Neurotol..

[44] Huyett P., Duvvuri U., Ferris R.L., Johnson J.T., Schaitkin B.M., Kim S. (2018). Perineural Invasion in Parotid Gland Malignancies. Otolaryngol. Head Neck Surg..

[45] Singh F.M., Mak S.Y., Bonington S.C. (2015). Patterns of spread of head and neck adenoid cystic carcinoma. Clin. Radiol..

[46] Foote M., Porceddu S., Poulsen M., Gorayski P. (2016). The Role of Postoperative Radiotherapy for Large Nerve Perineural Spread of Cancer of the Head and Neck. J. Neurol. Surg. Part B Skull Base.

[47] Amit M., Eran A., Billan S., Fridman E., Na’ara S., Charas T., Gil Z. (2016). Perineural Spread in Noncutaneous Head and Neck Cancer: New Insights into an Old Problem. J. Neurol. Surg. B Skull Base.

[48] Dijkema T., Raaijmakers C.P., Ten Haken R.K., Roesink J.M., Braam P.M., Houweling A.C., Moerland M.A., Eisbruch A., Terhaard C.H. (2010). Parotid gland function after radiotherapy: The combined michigan and utrecht experience. Int. J. Radiat. Oncol. Biol. Phys..

[49] Ling D.C., Kabolizadeh P., Heron D.E., Ohr J.P., Wang H., Johnson J., Kubicek G.J. (2015). Incidence of hospitalization in patients with head and neck cancer treated with intensity-modulated radiation therapy. Head Neck.

[50] Trotti A., Bellm L.A., Epstein J.B., Frame D., Fuchs H.J., Gwede C.K., Komaroff E., Nalysnyk L., Zilberberg M.D. (2003). Mucositis incidence, severity and associated outcomes in patients with head and neck cancer receiving radiotherapy with or without chemotherapy: A systematic literature review. Radiother. Oncol..

[51] Rong X., Tang Y., Chen M., Lu K., Peng Y. (2012). Radiation-induced cranial neuropathy in patients with nasopharyngeal carcinoma. A follow-up study. Strahlenther. Onkol..

[52] Dong Y., Ridge J.A., Ebersole B., Li T., Lango M.N., Churilla T.M., Donocoff K., Bauman J.R., Galloway T.J. (2019). Incidence and outcomes of radiation-induced late cranial neuropathy in 10-year survivors of head and neck cancer. Oral Oncol..

[53] Holliday E.B., Garden A.S., Rosenthal D.I., Fuller C.D., Morrison W.H., Gunn G.B., Phan J., Beadle B.M., Zhu X.R., Zhang X. (2015). Proton Therapy Reduces Treatment-Related Toxicities for Patients with Nasopharyngeal Cancer: A Case-Match Control Study of Intensity-Modulated Proton Therapy and Intensity-Modulated Photon Therapy. Int. J. Part. Ther..

[54] Bhattasali O., Holliday E., Kies M.S., Hanna E.Y., Garden A.S., Rosenthal D.I., Morrison W.H., Gunn G.B., Fuller C.D., Zhu X.R. (2016). Definitive proton radiation therapy and concurrent cisplatin for unresectable head and neck adenoid cystic carcinoma: A series of 9 cases and a critical review of the literature. Head Neck.

[55] Jensen A.D., Nikoghosyan A.V., Poulakis M., Höss A., Haberer T., Jäkel O., Münter M.W., Schulz-Ertner D., Huber P.E., Debus J. (2015). Combined intensity-modulated radiotherapy plus raster-scanned carbon ion boost for advanced adenoid cystic carcinoma of the head and neck results in superior locoregional control and overall survival. Cancer.

[56] Jensen A.D., Poulakis M., Nikoghosyan A.V., Welzel T., Uhl M., Federspil P.A., Freier K., Krauss J., Höss A., Haberer T. (2016). High-LET radiotherapy for adenoid cystic carcinoma of the head and neck: 15 years’ experience with raster-scanned carbon ion therapy. Radiother. Oncol..

[57] Ikawa H., Koto M., Takagi R., Ebner D.K., Hasegawa A., Naganawa K., Takenouchi T., Nagao T., Nomura T., Shibahara T. (2017). Prognostic factors of adenoid cystic carcinoma of the head and neck in carbon-ion radiotherapy: The impact of histological subtypes. Radiother. Oncol..

[58] Lang K., Adeberg S., Harrabi S., Held T., Kieser M., Debus J., Herfarth K. (2021). Adenoid cystic Carcinoma and Carbon ion Only irradiation (ACCO): Study protocol for a prospective, open, randomized, two-armed, phase II study. BMC Cancer.

[59] Huber P.E., Debus J., Latz D., Zierhut D., Bischof M., Wannenmacher M., Engenhart-Cabillic R. (2001). Radiotherapy for advanced adenoid cystic carcinoma: Neutrons, photons or mixed beam?. Radiother. Oncol..

[60] Prott F.J., Micke O., Haverkamp U., Willich N., Schüller P., Pötter R. (2000). Results of fast neutron therapy of adenoid cystic carcinoma of the salivary glands. Anticancer Res..

[61] Xu M.J., Wu T.J., van Zante A., El-Sayed I.H., Algazi A.P., Ryan W.R., Ha P.K., Yom S.S. (2018). Mortality risk after clinical management of recurrent and metastatic adenoid cystic carcinoma. J. Otolaryngol. Head Neck Surg..

[62] McDonald M.W., Zolali-Meybodi O., Lehnert S.J., Estabrook N.C., Liu Y., Cohen-Gadol A.A., Moore M.G. (2016). Reirradiation of Recurrent and Second Primary Head and Neck Cancer With Proton Therapy. Int. J. Radiat. Oncol. Biol. Phys..

[63] Seidensaal K., Harrabi S.B., Uhl M., Debus J. (2020). Re-irradiation with protons or heavy ions with focus on head and neck, skull base and brain malignancies. Br. J. Radiol..

[64] Jensen A.D., Poulakis M., Nikoghosyan A.V., Chaudhri N., Uhl M., Münter M.W., Herfarth K.K., Debus J. (2015). Re-irradiation of adenoid cystic carcinoma: Analysis and evaluation of outcome in 52 consecutive patients treated with raster-scanned carbon ion therapy. Radiother. Oncol..

[65] Dautruche A., Bolle S., Feuvret L., Le Tourneau C., Jouffroy T., Goudjil F., Zefkili S., Nauraye C., Rodriguez J., Herman P. (2018). Three-year results after radiotherapy for locally advanced sinonasal adenoid cystic carcinoma, using highly conformational radiotherapy techniques proton therapy and/or Tomotherapy. Cancer Radiother..

[66] Vischioni B., Dhanireddy B., Severo C., Bonora M., Ronchi S., Vitolo V., Fiore M.R., D’Ippolito E., Petrucci R., Barcellini A. (2020). Reirradiation of salivary gland tumors with carbon ion radiotherapy at CNAO. Radiother. Oncol..

[67] Sayan M., Vempati P., Miles B., Teng M., Genden E., Demicco E.G., Misiukiewicz K., Posner M., Gupta V., Bakst R.L. (2016). Adjuvant Therapy for Salivary Gland Carcinomas. Anticancer Res..

[68] Schoenfeld J.D., Sher D.J., Norris C.M., Haddad R.I., Posner M.R., Balboni T.A., Tishler R.B. (2012). Salivary gland tumors treated with adjuvant intensity-modulated radiotherapy with or without concurrent chemotherapy. Int. J. Radiat. Oncol. Biol. Phys..

[69] Ha H., Keam B., Ock C.Y., Kim T.M., Kim J.H., Chung E.J., Kwon S.K., Ahn S.H., Wu H.G., Sung M.W. (2021). Role of concurrent chemoradiation on locally advanced unresectable adenoid cystic carcinoma. Korean J. Intern. Med..

[70] Matsuba H.M., Spector G.J., Thawley S.E., Simpson J.R., Mauney M., Pikul F.J. (1986). Adenoid cystic salivary gland carcinoma. A histopathologic review of treatment failure patterns. Cancer.

[71] Sahara S., Herzog A.E., Nör J.E. (2021). Systemic therapies for salivary gland adenoid cystic carcinoma. Am. J. Cancer Res..

[72] Bobbio A., Copelli C., Ampollini L., Bianchi B., Carbognani P., Bettati S., Sesenna E., Rusca M. (2008). Lung metastasis resection of adenoid cystic carcinoma of salivary glands. Eur. J. Cardiothorac. Surg..

[73] Girelli L., Locati L., Galeone C., Scanagatta P., Duranti L., Licitra L., Pastorino U. (2017). Lung metastasectomy in adenoid cystic cancer: Is it worth it?. Oral. Oncol..

[74] Franzese C., Badalamenti M., Teriaca A., De Virgilio A., Mercante G., Cavina R., Ferrari D., Santoro A., Spriano G., Scorsetti M. (2021). Metastasis-directed stereotactic body radiation therapy in the management of oligometastatic head and neck cancer. J. Cancer Res. Clin. Oncol..

[75] Kobayashi D., Abe T., Saitoh J.I., Oike T., Sato H., Musha A., Mizukami T., Shimizu T., Nakano T., Ohno T. (2021). Stereotactic body radiotherapy for adenoid cystic carcinoma metastatic to the lung: A case report. J. Med. Case Rep..

